# A meta-analysis on the factors of postoperative delirium in patients undergoing routine care in the recovery room

**DOI:** 10.3389/fmed.2026.1795963

**Published:** 2026-03-27

**Authors:** Fenfen Zhang, Chenxuan Liu, Manjia Yang, Xue Bai, Bin Yang, Jiajia Yu

**Affiliations:** 1 Department of Anesthesiology (Operating Room), Yantai Affiliated Hospital of Binzhou Medical University, Yantai, Shandong, China; 2 School of Pharmacy, Yantai Campus, Binzhou Medical University, Yantai, Shandong, China; 3 Operating Room, Binzhou Medical University Hospital, Binzhou, Shandong, China; 4 Post-Anesthesia Care Unit, Binzhou Medical University Hospital, Binzhou, Shandong, China; 5 Department of Nursing, Yantai Affiliated Hospital of Binzhou Medical University, Yantai, Shandong, China

**Keywords:** meta-analysis, post-anesthesia care unit, postoperative delirium, recovery room, risk factors

## Abstract

**Objective:**

To systematically evaluate the risk factors associated with postoperative delirium (POD) in patients receiving routine care in the post-anesthesia care unit (PACU).

**Methods:**

Relevant literature concerning risk factors for POD in PACU patients was retrieved from CNKI, Wanfang, and VIP databases from inception up to August 2025. Three researchers independently completed literature screening, data extraction, quality evaluation, and evidence grading. Meta-analysis was performed using RevMan 5.3 software, and publication bias was assessed using Begg's test in Stata 18.0.

**Results:**

A total of 20 case-control studies involving 6,877 patients were included. Meta-analysis showed that risk factors for POD in PACU patients included: gender (OR = 1.17, 95%CI:1.03–1.33, *P* = 0.02), age (SMD = 2.05, 95%CI:1.52–2.57, *P* < 0.00001), prolonged operation time (SMD = 1.02, 95%CI:0.44–1.60, *P* = 0.0006), low BMI (SMD = −0.29, 95%CI:−0.53–0.04, *P* = 0.02), prolonged anesthesia time (SMD = 1.58, 95%CI:0.88–2.88, *P* < 0.00001), hypertension (OR = 1.27, 95%CI:1.06–1.51, *P* = 0.009), diabetes mellitus (OR = 1.44, 95%CI:1.01–2.06, *P* = 0.04), smoking (OR = 1.38, 95%CI:1.11–1.71, *P* = 0.004), decreased hemoglobin level (SMD = −1.05, 95%CI: −3.97–0.12, *P* = 0.04), alcohol consumption (OR = 1.33, 95%CI:1.05–1.63, *P* = 0.02), increased intraoperative blood loss (SMD = 3.74, 95%CI:1.60–5.87, *P* = 0.0006), higher American Society of Anesthesiologists (ASA) physical status classification (OR = 0.66, 95%CI:0.47–0.92, *P* = 0.02), insufficient use of dexmedetomidine (OR = 0.33, 95%CI:0.23–0.52, *P* < 0.00001), prolonged hospital stay (SMD = 2.01, 95%CI:0.76–3.27, *P* = 0.002), low educational level (OR = 1.43, 95%CI:1.03–1.98, *P* = 0.03), history of cerebral infarction (OR = 2.31, 95%CI:1.48–3.60, *P* = 0.0002), and decreased postoperative albumin level (SMD = −1.91, 95%CI: −2.76–1.07, *P* < 0.00001).

**Conclusion:**

Multiple demographic, perioperative, and disease-related factors are associated with the occurrence of POD in PACU patients under routine care. This study provides an evidence-based reference for the early identification and prevention of POD. The clinical value of these risk factors as an intervention still needs to be verified by high-quality prospective studies.

**Systematic trial registration:**

https://www.crd.york.ac.uk/PROSPERO/view/CRD420261341102, identifier: CRD420261341102

## 1 Introduction

Postoperative delirium (POD) is an acute and fluctuating cerebral dysfunction syndrome that usually occurs within 24–72 h after surgery, characterized by impaired consciousness and cognitive dysfunction. It has an acute onset and fluctuating clinical course, and is closely associated with postoperative prognosis.

As a common postoperative complication, POD may lead to incomplete recovery of cognitive function and subsequent persistent cognitive impairment. It also reduces patients' mobility and treatment compliance, increasing the risk of adverse nursing events. In severe cases, respiratory depression and weakened airway protection may result in serious complications such as pulmonary infection and respiratory failure.

Intrinsic factors vary among individuals and provide a biological basis for POD. With aging, the number of cerebral neurons and synaptic connectivity gradually decrease, accompanied by reduced compensatory capacity against ischemia and inflammation, as well as impaired vasomotor regulation. Hypertension, diabetes mellitus, and other chronic diseases also serve as important intrinsic risk factors. In addition to these internal conditions, extrinsic factors including operation duration, anesthesia time, and dexmedetomidine administration, also contribute to the development of POD. Delayed identification and intervention may prolong hospital stay and increase complication rates, highlighting the importance of early detection and timely management.

However, most current studies on POD risk factors have focused on patients in general wards or intensive care units, whereas evidence specifically targeting post-anesthesia care unit (PACU) patients—who present residual anesthetic effects and unstable consciousness—remains limited.

The PACU is a critical clinical unit for postoperative awakening, vital sign monitoring, and early prevention and treatment of postoperative complications (1). Patients in the PACU are often affected by residual anesthetic drugs and disturbed consciousness, placing them at high risk of adverse events. Therefore, the PACU represents a key window for early intervention of postoperative complications.

Although the adverse impacts of POD are well recognized, high-quality research on POD in the PACU—the golden window for early recognition—is still insufficient. Previous studies have mainly focused on patients in general wards or ICUs, often ignoring the unique clinical features of PACU patients, such as residual anesthesia and fluctuating levels of consciousness. Furthermore, most available studies are single-centered with small sample sizes (mostly < 400 cases), leading to considerable heterogeneity in risk factor analysis. For example, the relationship between prolonged operation time (e.g., >2.5 h) and POD risk remains controversial. These inconsistencies have limited the establishment of standardized, evidence-based strategies for preventing and managing PODs in the PACU.

With specialized monitoring teams and comprehensive emergency equipment, the PACU provides an irreplaceable setting for early detection and intervention of POD. Therefore, systematically exploring the risk factors of POD in PACU patients is clinically important for optimizing perioperative care. In this meta-analysis, we synthesized evidence from published single-center and multi-center studies to identify key risk factors of POD in PACU patients. The results may help guide targeted nursing strategies, promote early detection and intervention of POD, and ultimately improve the postoperative quality of life of patients.

## 2 Materials and methods

### 2.1 Literature search strategy

This study was designed in strict accordance with the PICOS framework.

Participants (P): postoperative patients admitted to the PACU who developed POD;

Outcomes (O): risk factors associated with POD;

Study design (S): case-control studies and retrospective cohort studies (2).

Computer searches were performed in the Wanfang Data, China National Knowledge Infrastructure (CNKI), and VIP Chinese Science and Technology Journal Database. The retrieval period was from the establishment of each database to August 30, 2025. A combination of subject terms and free words was used to ensure comprehensive retrieval. Subject headings were matched with the thesauri of Wanfang Data, MeSH, and SinoMed.

Chinese search terms: postoperative delirium, anesthesia, nursing, and recovery room.

English search terms: recovery room, delirium, risk factor, and postoperative complications.

Taking Web of Science as an example, the detailed search strategy is shown in Table 1.

**Table 1:** Search strategy of web of science.

**Steps**	**Search strategy**
#1	TS = (“Post-Anesthesia Care Unit” OR “PACU” OR “surgery” OR “anesthesia” OR “Preoperative”)
#2	TS = (“POD” OR “Postoperative Delirium” OR “delirium” OR “hyperactive” OR “del”)
#3	TS = (“risk factor” OR “potential factor” OR “influencing factor”)
#4	#1 AND #2 AND #3 and Preprint Citation Index (exclude database)

### 2.2 Inclusion and exclusion criteria

Inclusion criteria:

1. Study population: patients diagnosed with postoperative delirium (POD) based on: ① Internationally or domestically recognized scales (e.g., Confusion Assessment Method [CAM], Confusion Assessment Method for the Intensive Care Unit [CAM-ICU], and Nursing Delirium Screening Scale [Nu-DESC]); ② Diagnostic criteria formulated by the Chinese Medical Association and its professional branches (3); ③ Other clinically validated POD diagnostic criteria (4).
2. Study content: studies investigating POD-related factors (e.g., gender, age, operation duration, BMI, anesthesia time, hypertension, diabetes mellitus, and smoking status).
3. Language: Chinese or English.
4. Study design: case-control studies or retrospective cohort studies.

Exclusion criteria:

1. Patients with preoperatively diagnosed delirium.
2. Patients with definitive, independent POD triggers irrelevant to the study (e.g., intracranial infection, severe preoperative mental illness).
3. Literature published more than 5 years prior to the search date.
4. Reviews, abstracts, conference proceedings, or duplicate publications.
5. Literature with unavailable full text, inaccessible original data, or excessively small sample sizes.

### 2.3 Literature selection and data extraction

The literature was imported into the NoteExpress software. Two researchers independently performed literature screening and data extraction, with discrepancies resolved through consultation with a third researcher. The screening process included: ① Removing duplicate studies via software; ② Initial screening based on titles and abstracts to exclude irrelevant literature; ③ Full-text review of remaining studies to confirm eligibility and extract data. Extracted data included:

1. Author name, publication year, and country;
2. Total sample size and subgroup sizes (cases/controls);
3. POD-related factors (e.g., age, gender, hypertension, diabetes mellitus, operation duration, and anesthesia time);
4. Effect sizes (SMD, OR) and corresponding 95% confidence intervals (CIs);
5. POD diagnostic criteria and complication types.

### 2.4 Literature quality evaluation

Two researchers independently assessed the quality of included studies, with disagreements adjudicated by a third researcher. The Newcastle-Ottawa Scale (NOS) (5) was used to evaluate case-control and retrospective cohort studies, covering three dimensions: study population selection, group comparability, and exposure/outcome measurement. The NOS scale has a total score of 9 points, with studies classified as: low quality (0–4 points), moderate quality (5–6 points), and high quality (7–9 points).

### 2.5 Statistical analysis

Data were preprocessed using Excel and imported into RevMan 5.3 software for meta-analysis. For data presented as medians (incompatible with direct meta-analysis), a conversion tool developed by the Department of Mathematics, Hong Kong Baptist University (https://www.math.hkbu.edu.hk/~tongt/papers/median2mean.html) was used to convert medians to means (6, 7). Heterogeneity was assessed using the I^2^ statistic and Q-test: a fixed-effects model was applied if *I*^2^ ≤ 50% and *P* ≥ 0.1 (no significant heterogeneity); otherwise, a random-effects model was used.

Standardized mean differences (SMDs) were used to express effect sizes for continuous variables, and odds ratios (ORs) for dichotomous variables. Sensitivity analysis was performed for factors with ≥5 included studies and *I*^2^ ≥ 50%. The Cochrane Risk of Bias tool was used to assess potential biases, and a quality assessment plot was generated to evaluate publication bias. Statistical significance was set at *P* < 0.05.

### 2.6 Evidence evaluation

The GRADE (Grading of Recommendations Assessment, Development and Evaluation) tool (8) was used to evaluate the quality of evidence, considering five domains: risk of bias, indirectness, publication bias, inconsistency, and imprecision. Evidence quality was graded as high (A), moderate (B), low (C), or very low (D). High quality indicates no downgrading; moderate quality indicates one level of downgrading; very low quality indicates three levels of downgrading.

## 3 Results

### 3.1 Literature search and screening results

**Figure 1:**
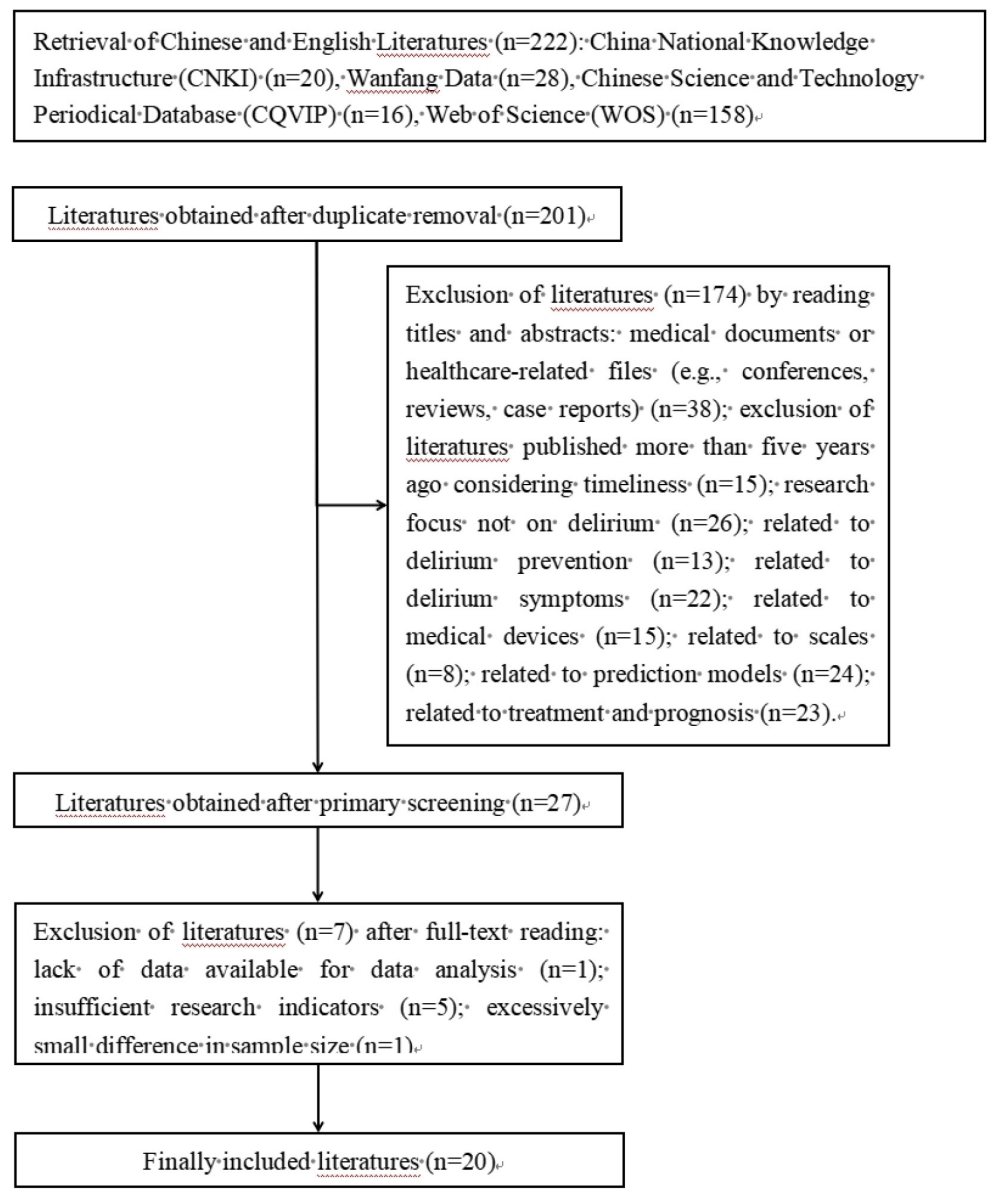
The flow chart of literature screening.

A total of 222 studies were retrieved through the systematic literature search. After removing 21 duplicate records using NoteExpress software, 201 studies were excluded based on title and abstract screening due to irrelevance to the research topic or non-compliance with study design requirements. This left 27 studies for full-text review. Following rigorous evaluation against the predefined inclusion and exclusion criteria, 7 studies were further excluded (3 with unavailable full text, 2 with insufficient data, and 2 not meeting the diagnostic criteria for POD). Finally, 20 eligible studies (9–28) were included in the meta-analysis, consisting of 17 Chinese studies (9–25) and 3 English studies (26–28). The detailed literature retrieval and screening process is illustrated in Figure 1.

### 3.2 Basic characteristics and quality evaluation of included literatures

The 20 included studies had a total sample size of 6,877 patients, including 1,271 POD cases and 5,606 non-POD controls. All studies were conducted in China with a case-control design, and each clearly specified the diagnostic criteria for POD (29–37) (e.g., CAM scale, CAM-ICU scale, or criteria from the Chinese Medical Association).

Literature quality was assessed using the Newcastle-Ottawa Scale (NOS). Among the 20 studies (9–28), 17 were rated as high-quality (NOS score 7–9 points) (9–11, 13–20, 23–28), and 3 as moderate-quality (NOS score 5–6 points) (12, 21, 22). No low-quality studies (NOS score 0–4 points) were included. The basic characteristics (e.g., author, publication year, sample size, and POD diagnostic criteria) and quality evaluation results of the included studies are summarized in Table 2.

**Table 2:** The specific characteristics and quality evaluation of the included literature.

**First author**	**Year of publication**	**Delirium diagnostic criteria**	**Sample size (delirium/non-delirium)**	**Exposure factor**	**NOS score (points)**
Xiangxiang Liu	2023	CAM	100 (39/61)	1, 2, 3, 4, 5, 6,	7
Xinlin Li	2021	SAS, SDS	200 (83/200)	1, 2, 4, 7, 8, 9, 10, 11, 12, 13, 14,	8
Laixiu Yan	2024	CAM	116 (29/87)	1, 2, 4, 5, 7, 8, 11, 12, 15, 16, 17, 18, 19, 20, 21, 22, 23, 24, 25, 26	8
Linsong Yuan	2023	PAED	512 (120/392)	1, 2, 7, 14, 24, 27, 28, 29, 30	8
He, Chuan	2024	CAMS	100 (10/90)	1, 2, 4, 5, 7, 8, 12, 15, 17, 18, 19, 20, 22, 25, 29, 31, 32, 33	8
Yang Bai	2025	RASS, CAM—ICU, 3D-CAM	260 (88/172)	1, 2, 6, 7, 8, 15, 17, 23, 24, 26, 27, 31, 32, 33	8
Shangkun Liu	2021	CAM—ICU	228 (57/171)	1, 2, 7, 8, 12, 13, 21, 33, 34,	8
Yalan Chen	2025	Nu—DESC	1 851 (1 738/113)	1, 2, 7, 32, 34,	6
Yulian Yang	2020	Nu—DESC	300 (64/236)	1, 2, 7, 8, 24, 35,	6
Mansencal Nicolas	2025	CAM	451 (81/370)	1, 2, 15, 17, 18, 32, 36, 37	7
Jiao Ma	2024	CCMD-3	80 (34/46)	1, 2, 4, 5, 7, 8, 15, 17, 18, 24, 32,	8
Xiaoyan Chen	2024	CAM—ICU	133 (42/91)	1, 2, 15, 38, 39	7
Wenzhe Li	2025	DRS-R-98	310 (85/225)	1, 2, 3, 4, 5, 15, 17, 31, 32, 36,	8
Xiufang Tang	2025	CAM	385 (51/334)	1, 2, 3, 4, 5, 6, 7, 12, 33	8
Weiping Zhang	2024	DSM—IV	566 (41/525)	1, 2, 3, 4, 5, 7, 12, 15, 17, 19, 20, 23, 37,	8
Hanming Li	2024	CAM	252 (31/221)	1, 2, 4, 5, 6, 7, 8, 12, 15, 17, 19, 23, 24	8
Xiufeng Xie	2024	CAM	597 (131/466)	1, 15, 17,	6
Yuedong Zhang	2024	DSM-−5	108 (42/66)	1, 3, 4, 5, 7, 8, 24, 32, 33	7
Jin Yi	2024	DSM-−5	172 (83/89)	1, 2, 4, 5, 6, 7, 12, 15, 26, 39	7
Qiang Sun	2023	CAM	156 (35/121)	1, 2, 7, 15, 17, 23, 31, 33	7

CAM, confusion assessment method; SAS, Zung self-rating anxiety scale; SDS, self-rating depression scale; PAED, pediatric anesthesia emergence delirium scale; CAMS, confusion assessment method for severity; RASS, richmond agitation-sedation scale; CAM-ICU, confusion assessment method for the intensive care unit; 3D-CAM, 3-min confusion assessment method; Nu-DESC, nursing delirium screening scale. The exposure factors included: 1. Age; 2. Gender; 3. Educational Level; 4. Smoking; 5. Alcohol Consumption; 6. Dexmedetomidine; 7. Operation Time; 8. BMI (Body Mass Index); 9. SAS Score; 10. Total Protein; 11. Blood Glucose; 12. Hemoglobin; 13. Hematocrit; 14. Blood Loss; 15. Hypertension; 16. Hyperlipidemia; 17. Diabetes Mellitus; 18. Length of Hospital Stay; 19. Albumin; 20. Platelet; 21. Total Cholesterol; 22. Triglyceride; 23. Blood Loss Volume; 24. Anesthesia Time; 25. Mean Arterial Pressure (MAP); 26. Fluid Infusion Volume; 27. Total Dose of Sufentanil; 28. Extubation Time; 29. Blood Oxygen Saturation (SpO_2_); 30. Length of Stay; 31. Cerebral Infarction; 32. Coronary Heart Disease; 33. ASA Physical Status Classification; 34. Surgical Site; 35. PACU Length of Stay; 36. Hypoglycemia; 37. Opioid Use; 38. Deep Sedation; 39. Mechanical.

### 3.3 Meta-analysis results

#### 3.3.1 Effect of smoking on postoperative delirium in PACU patients

A total of 11 studies (9–11, 13, 14, 16, 18, 23–25, 27) involving 2,389 patients were included to analyze the association between smoking and postoperative delirium (POD) in PACU patients receiving routine care. Heterogeneity testing indicated low statistical heterogeneity among the included studies (*I*^2^ = 26%, *P* = 0.19), so a fixed-effects model was adopted for meta-analysis.

**Figure 2:**
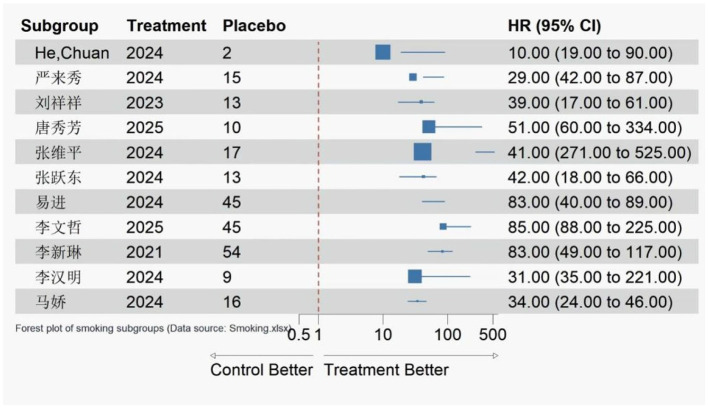
Forest plot of the effect of smoking on postoperative delirium in patients receiving routine post-anesthesia care unit (PACU) care.

The results showed that smoking was significantly associated with an increased risk of POD in PACU patients [OR = 1.38, 95%CI (1.11, 1.71), *P* = 0.004]. This suggests that smokers have a 38% higher risk of developing POD compared with non-smokers. The forest plot for the meta-analysis of smoking and POD risk is shown in Figure 2.

#### 3.3.2 Heterogeneity analysis and exploration of influencing factors

Preliminary meta-analysis using RevMan 5.3 software revealed high statistical heterogeneity (*I*^2^ > 50%) among the included studies for 17 potential influencing factors of POD: gender, age, hypertension, operation time, diabetes mellitus, BMI, alcohol consumption, intraoperative blood loss, anesthesia time, ASA physical status classification, educational level, hospital stay, history of cerebral infarction, dexmedetomidine use, hemoglobin level, and postoperative albumin concentration. To clarify the source of heterogeneity, additional exploratory analyses were performed (e.g., subgroup analysis based on study quality, sample size, or diagnostic criteria; sensitivity analysis for factors with ≥5 included studies).

#### 3.3.3 Comprehensive meta-analysis results of POD influencing factors

After adjusting for heterogeneity and validating result robustness, meta-analysis confirmed that 17 factors were significantly associated with the risk of POD in PACU patients receiving routine care. These factors included:

Demographic and baseline characteristics: advanced age, male gender, low educational level, history of cerebral infarction, hypertension, diabetes mellitus, smoking history, and alcohol consumption history;

Perioperative indicators: prolonged operation time, prolonged anesthesia time, large intraoperative blood loss, and higher ASA physical status classification (≥ III);

Anthropometric and prognostic indicators: low BMI, prolonged hospital stay.

All statistically significant effect sizes (OR/SMD values, 95% CIs, and *P* values) for the meta-analysis of POD influencing factors in PACU patients are summarized in Table 3.

**Table 3:** Results of meta-analysis on factors for postoperative delirium in patients undergoing routine post-anesthesia care unit (PACU) nursing.

**Factor**	**Included studies (n)**	**Sample size of patients**	Heterogeneity test		Meta-analysis results			
			***I**^2^* **(%)**	***P*** **value**	**Effect size**	**95%CI**	**Effect model**	***P*** **value**
Gender	20	6,877	46	0.01	OR = 1.17	1.03~1.33	Fixed	0.02
Age	19	6,769	98	< 0.000 01	SMD = 2.05	1.52~2.57	Random	< 0.000 01
Hypertension	13	3,291	28	0.16	OR = 1.27	1.06~1.51	Fixed	0.009
Operation time	12	2,367	97	< 0.000 01	SMD = 1.02	0.44~1.60	Random	0.000 6
Diabetes mellitus	11	2,925	53	0.02	OR = 1.44	1.01~2.06	Random	0.04
Smoking	11	2, 389	26	0.19	OR = 1.38	1.11~1.71	Fixed	0.004
Alcohol consumption	10	2,189	0	0.70	OR = 1.33	1.05~1.63	Fixed	0.02
BMI	10	1,649	80	< 0.000 01	SMD = −0.29	−0.53 ~−0.04	Random	0.02
Anesthesia time	5	1,064	96	< 0.000 01	SMD = 1.58	0.88~2.28	Random	< 0.000 01
ASA physical Status classification	5	1,009	9	0.35	OR = 0.66	0.47~0.92	Fixed	0.02
Educational level	5	903	59	0.05	OR = 1.43	1.03~1.98	Fixed	0.03
Blood loss	5	776	99	< 0.000 01	SMD = 3.74	1.60~5.87	Random	0.000 6
Length of hospital stay	4	893	98	< 0.000 01	SMD = 2.01	0.76~3.27	Random	0.002
Cerebral Infarction	4	826	0	0.80	OR = 2.31	1.48~3.60	Fixed	0.000 2
Dexmedetomidine	3	612	10	0.33	OR = 0.35	0.23~0.52	Fixed	< 0.000 01
Hemoglobin	3	412	97	< 0.000 01	SMD = −1.05	−3.97 ~−0.12	Random	0.04
Postoperative albumin	2	818	90	0.002	SMD = −1.91	−2.76 ~−1.07	Random	< 0.000 01

### 3.4 Sensitivity analysis

A one-study-at-a-time exclusion approach was employed to perform sensitivity analysis for POD-related factors with high initial heterogeneity (*I*^2^ > 50%).

The results demonstrated that the statistical heterogeneity of five factors—BMI, diabetes mellitus, intraoperative blood loss, educational level, and hospital stay—decreased significantly after sequentially excluding individual studies, indicating that the combined effect sizes of these factors were susceptible to the influence of specific included studies. In contrast, the heterogeneity of age, operation time, and anesthesia time remained largely unchanged throughout the exclusion process, confirming the high stability and credibility of the meta-analysis results for these three factors.

Notably, only 2–3 studies reported data on hemoglobin levels and postoperative albumin concentrations, which was insufficient to apply the one-by-one exclusion method. Therefore, sensitivity analysis was not conducted for these two factors. Detailed results of the sensitivity analysis are presented in Table 4.

**Table 4:** Sensitivity analysis of factors influencing postoperative delirium in patients receiving routine post-anesthesia care unit (PACU) nursing.

**Risk factor**	***I^2^*value**	***P* value**	**SMD (OR) Value (95%CI)**
Age	96	< 0.000 01	1.25 (0.80, 1.69)
Operation time	92	< 0.000 01	0.60 (0.20, 1.00)
BMI	0	0.39	−0.05 (−0.17, 0.07)
Anesthesia time	92	< 0.000 01	1.15 (0.56, 1.75)
Diabetes mellitus	0	0.09	1.22 (0.97, 1.53)^a^
Blood loss	0	0.96	0.00 (−0.17, 0.16)
Educational level	0	0.80	1.05 (0.72, 1.54)^a^
Length of hospital stay	37	0.21	3.03 (2.66, 3.40)

^a^indicates the Odds Ratio (OR) value.

### 3.5 Publication bias analysis

**Figure 3:**
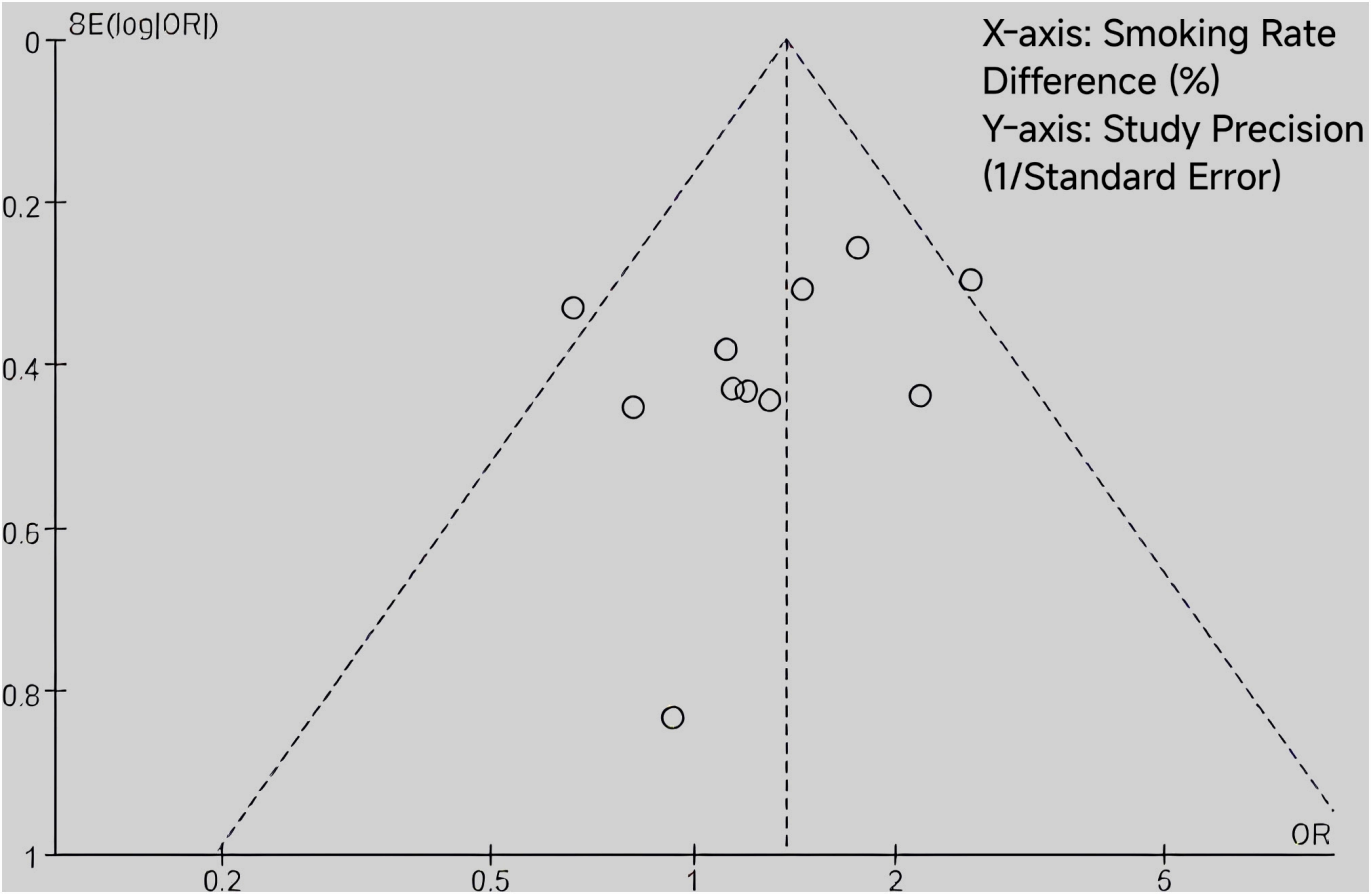
Funnel plot of the effect of smoking on postoperative delirium in patients undergoing routine post-anesthesia care unit (PACU) care.

**Figure 4:**
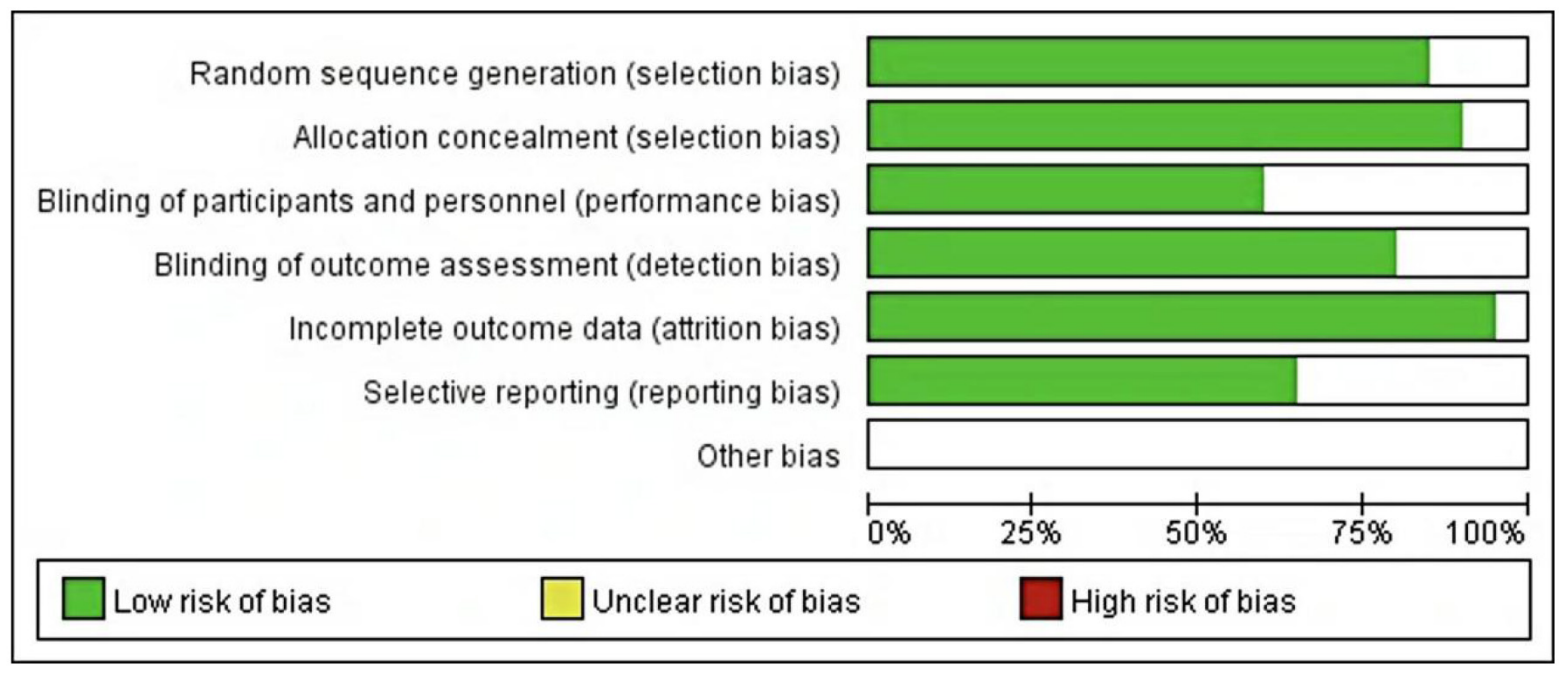
Proportion of each quality evaluation item of included literature.

**Figure 5:**
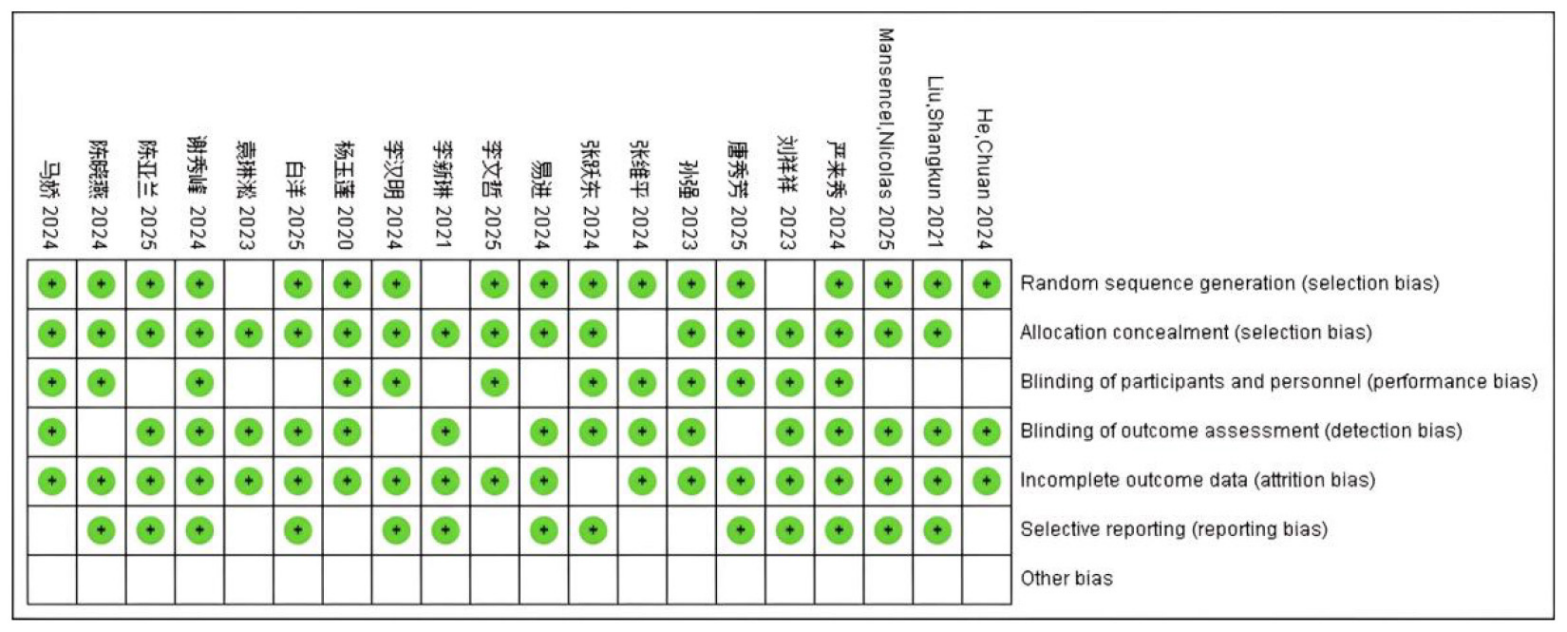
Schematic diagram of quality evaluation of included literature.

When the number of research literatures was no less than 5, funnel plot method was used for publication bias evaluation. Taking gender as an example, the funnel plot showed that most studies were evenly distributed on both sides of the average effect (Figure 3). Begg's test was performed to further assess publication bias, and the results showed no significant publication bias (*P* > 0.05), indicating the reliability of the meta-analysis results. The funnel plot for publication bias assessment is shown in Figure 3. The risk of bias graph and risk of bias summary are presented in Figure 4 and Figure 5, respectively.

### 3.6 Evidence evaluation

The GRADE evidence evaluation of the factors of postoperative delirium in patients receiving routine care in the recovery room is shown in Table 5. There were 9 medium-quality (B) factors and 9 low-quality (C) factors. The reason for the downgrading of bias risk was that no appropriate method was used for the experiment and single variable was not controlled.

1. Risk of bias downgrading: most included studies were observational (case-control studies) with inadequate control of confounding factors (e.g., lack of adjustment for multiple covariates), leading to a high risk of selection and measurement bias.
2. Imprecision downgrading: wide 95% confidence intervals for some factors, small total sample size (< 1,500 participants) for individual outcomes, and increased random error resulting in unstable effect estimates.
3. Inconsistency downgrading: significant statistical heterogeneity (I^2^≥75%) among included studies, with inconsistent effect directions or magnitudes that could not be explained by subgroup analyses (e.g., study quality, sample size).

**Table 5:** Evidence quality evaluation of risk factors for delirium in patients receiving routine post-anesthesia care unit (PACU) care.

**Risk factor**	Evidence quality assessment					**Evidence quality**	**Evidence grade (level)**
	**Risk of bias**	**Imprecision**	**Inconsistency**	**Indirectness**	**Publication bias**		
Gender	No significant risk of bias	No significant imprecision	No significant inconsistency	No significant indirectness	Not detected	High risk	A
Age	No significant risk of bias	No significant imprecision	No significant inconsistency	No significant indirectness	Not detected	High risk	A
Operation time	No significant risk of bias	No significant imprecision	No significant inconsistency	No significant indirectness	Not detected	High risk	A
BMI	No significant risk of bias	No significant imprecision	No significant inconsistency	No significant indirectness	Not detected	High risk	A
Hemoglobin	Significant^a^	Significant^b^	No significant inconsistency	No significant indirectness	Not detected	Low level	C
Anesthesia time	Significant^a^	No significant imprecision	No significant inconsistency	No significant indirectness	Not detected	Moderate quality	B
Hypertension	No significant risk of bias	No significant imprecision	No significant inconsistency	No significant indirectness	Not detected	High risk	A
Diabetes mellitus	No significant risk of bias	No significant imprecision	No significant inconsistency	No significant indirectness	Not detected	High risk	A
Smoking	No significant risk of bias	No significant imprecision	No significant inconsistency	No significant indirectness	Not detected	High risk	A
Cerebral infarction	Significant^a^	No significant imprecision	No significant inconsistency	No significant indirectness	Not detected	Moderate quality	B
Alcohol consumption	No significant risk of bias	No significant imprecision	No significant inconsistency	No significant indirectness	Not detected	High risk	A
Blood loss	Significant^a^	Significant^b^	No significant inconsistency	No significant indirectness	Not detected	Low level	C
ASA classification	Significant^a^	No significant imprecision	No significant inconsistency	No significant indirectness	Not detected	Moderate quality	B
Postoperative albumin	Significant^a^	No significant imprecision	Significant^c^	No significant indirectness	Not detected	Low level	C
Dexmedetomidine	Significant^a^	Significant^b^	No significant inconsistency	No significant indirectness	Not detected	Low level	C
Educational level	Significant^a^	No significant imprecision	No significant inconsistency	No significant indirectness	Not detected	Moderate quality	B
Length of hospital stay	Significant^a^	No significant imprecision	Significant^c^	No significant indirectness	Not detected	Low level	C

^a^Downgraded by one level for bias risk: some studies had problems such as lack of random methods; ^b^Downgraded by one level for imprecision: wide confidence interval due to too few included patients or observed events; ^c^Downgraded by one level for inconsistency: inconsistent results of different studies without reasonable explanation. A = High quality, B = Medium quality, C = Low quality.

## 4 Discussion

The post-anesthesia care unit (PACU) is a high-risk setting for postoperative delirium (POD), as patients in this phase are often affected by residual anesthetic effects, unstable vital signs, and physiological stress responses. Comprehensive assessment of physical and psychological status is essential in routine PACU care, and adherence to the principle of “early detection and timely intervention” can effectively reduce adverse outcomes once POD occurs.

Against the background of population aging in China, the number of elderly surgical patients has increased substantially. As advanced age is a well-recognized risk factor for POD, identifying risk factors and establishing targeted preventive strategies are of great clinical significance for improving the prognosis of elderly patients and optimizing perioperative management.

To our knowledge, this study is the first comprehensive meta-analysis to systematically quantify and synthesize risk factors for postoperative delirium specifically in patients undergoing routine care in the post-anesthesia care unit (PACU).

We identified 17 factors associated with POD, including demographic characteristics (age, gender, educational level), preoperative comorbidities (hypertension, diabetes mellitus, and cerebral infarction), lifestyle factors (smoking, alcohol consumption), perioperative indicators (operation time, anesthesia time, intraoperative blood loss, and ASA classification), clinical interventions (dexmedetomidine), anthropometric indicators (low BMI), and laboratory parameters (hemoglobin, postoperative albumin). These findings provide a reference for early risk screening and personalized intervention. It should be noted that most included studies were conducted in China, which may limit the generalizability of our findings to non-Chinese populations due to potential differences in clinical practices, demographic characteristics, and healthcare systems.

### 4.1 Demographic characteristics

Age and male gender were independent risk factors for POD, whereas educational level showed no significant independent association. Elderly patients often present with multiple chronic diseases, cognitive decline, and impaired immune function. Surgical stress further disrupts neurotransmitter regulation and increases susceptibility to POD (38). The higher risk in male patients may be related to a higher prevalence of smoking and alcohol consumption, as well as delayed detection of psychological distress due to a higher rate of living alone among elderly men.

The weak association between educational level and POD may reflect confounding by age. Since patients aged 65 years and above accounted for a large proportion in the POD group, the previously observed correlation may not represent a genuine causal relationship. In addition, low educational level is often related to poor comprehension of perioperative health guidance and insufficient ability to express physical discomfort, which may aggravate psychological anxiety and physiological stress in PACU, further increasing the risk of delirium.

Clinical care should focus on elderly and male patients as high-risk groups, with strengthened preoperative evaluation, close monitoring of consciousness and cognitive function, and early psychological intervention.

### 4.2 Preoperative comorbidities

Hypertension, diabetes mellitus, and a history of cerebral infarction were independent risk factors for POD. Diabetes may induce POD by impairing cerebral glucose metabolism and reducing brain regulatory capacity (39). An et al. (40) reported that hypertension is the most common cardio-cerebrovascular comorbidity in diabetic patients. Elevating mean systemic arterial pressure, imposes additional hemodynamic stress on vital organs. At the same time, diabetes can speed up hypertension progression: for example, hyperglycemia increases cross-linking of subendothelial collagen fibers, worsens vascular wall fibrosis, and in turn promotes hypertension development. Hypertension causes chronic cerebral microvascular damage and impairs cerebral perfusion. A history of cerebral infarction indicates structural brain injury, which further reduces brain tolerance to perioperative stress. Ma et al. (41) further observed that hypertensive patients exhibit a higher baseline risk for cognitive and mental disturbances, predisposing them to postoperative delirium (POD). Cerebral infarction, as a manifestation of acute cerebrovascular compromise, can directly precipitate acute mental dysfunction, which may subsequently progress to delirium. Patients with a prior history of cerebral infarction already have baseline brain damage; surgical stress adds to this burden, impairs cerebral autoregulation, and ultimately leads to POD. Notably, sensitivity analysis reduced heterogeneity related to diabetes, supporting the robustness of its association with POD. In contrast, hypertension and cerebral infarction showed low heterogeneity from the start. Sensitivity analysis also attenuated diabetes-related heterogeneity, further confirming the robustness of this association.

Notably, sensitivity analysis reduced heterogeneity for diabetes, supporting the robustness of its association with POD. By contrast, hypertension and cerebral infarction showed low initial heterogeneity.

For patients with these comorbidities, targeted perioperative management is necessary, including strict blood glucose and blood pressure control, optimized cerebral perfusion, and enhanced cognitive monitoring to reduce POD risk.

### 4.3 Intraoperative conditions

Neither operation duration nor anesthesia time was significantly associated with POD, despite suggestions from some individual studies. High heterogeneity was observed for both operation time (*I*^2^ = 97%) and anesthesia time (*I*^2^ = 96%); thus, random-effects models were applied. The 95% CIs for pooled effects included 1, indicating no independent effect on POD risk.

The inconsistency between our results and some previous studies may be due to differences in surgical types, anesthesia protocols, baseline characteristics, and POD diagnostic criteria. Confounding factors such as hemodynamic stability and analgesia management may also mediate the relationship between operation/anesthesia time and POD. Future research may focus on developing risk prediction models and targeted intervention strategies for the PACU population.

### 4.4 Other factors

Low BMI, smoking, alcohol consumption, ASA classification, intraoperative blood loss, hospital stay, dexmedetomidine use, low hemoglobin, and low postoperative albumin were associated with POD. Sensitivity analysis reduced heterogeneity for low BMI and intraoperative blood loss, supporting the reliability of these associations.

Low BMI was an independent risk factor for POD, mainly due to insufficient nutritional reserve and weakened tolerance to surgical stress. Smoking and long-term alcohol consumption damage vascular function and disrupt neurotransmitter balance, both contributing to POD.

Patients with POD had higher ASA classification. However, the pooled OR for ASA classification was 0.66, which appeared to suggest a protective effect. This paradoxical result is likely due to reverse causality or misclassification during data extraction. Clinically, higher ASA classification indicates more severe comorbidities and poorer physiological reserve, which should theoretically increase POD risk. Clinically, higher ASA classification indicates more severe comorbidities and poorer physiological reserve, which should theoretically increase POD risk (42). Further studies with standardized data collection are warranted.

Increased intraoperative blood loss may cause hypotension and cerebral hypoperfusion, thereby increasing POD risk. Dexmedetomidine exerts sedative and anxiolytic effects by stimulating α_2_ adrenergic receptors in the locus coeruleus, which helps prevent POD. For PACU patients with residual anesthetic effects and unstable consciousness, this drug can also regulate the balance of sympathetic and parasympathetic nerves, reduce the fluctuation of vital signs, and alleviate cerebral metabolic disorder caused by perioperative stress, thus exerting a protective effect on cognitive function. Low hemoglobin leads to cerebral hypoxia, and low postoperative albumin reflects poor nutritional status; both reduce brain repair capacity and tolerance to stress.

Clinical staff should monitor these indicators closely, conduct reasonable risk stratification using ASA classification, and intervene promptly when POD occurs to alleviate patient suffering.

## 5 Conclusion

In summary, multiple factors are associated with postoperative delirium in patients undergoing routine care in the post-anesthesia care unit, including male gender, older age, prolonged operation time, low BMI, prolonged anesthesia time, hypertension, diabetes mellitus, smoking, low hemoglobin level, alcohol consumption, increased intraoperative blood loss, higher ASA classification, insufficient dexmedetomidine use, prolonged hospital stay, low educational level, history of cerebral infarction, and low postoperative albumin level.

These findings provide an updated evidence-based synthesis of risk factors for postoperative delirium in the PACU setting.

Some potential influencing factors, such as total protein, hyperlipidemia, and extubation time, could not be fully analyzed due to the limited number of relevant studies.

Clinical nurses should pay close attention to these risk factors, identify high-risk patients early, and implement timely interventions to improve the prevention and management of postoperative delirium in the recovery room.

## 6 Limitations

Several limitations of this meta-analysis should be acknowledged.

First, only observational retrospective cohort and case-control studies were included, which inherently carry a higher risk of selection bias, confounding bias, and measurement bias. Most included studies did not perform multivariable adjustment, which may have affected the stability of the effect estimates.

Second, the number of included studies for certain risk factors was relatively small (e.g., postoperative albumin, hemoglobin), with high between-study heterogeneity that could not be fully explained by subgroup or sensitivity analyses. These issues may reduce the reliability and generalizability of the corresponding results.

Third, the diagnostic tools for postoperative delirium varied slightly across included studies, including CAM, CAM-ICU, Nu-DESC, and other clinical criteria, which may have introduced clinical heterogeneity and influenced the pooled results.

Fourth, most included studies were conducted in China, and most were single-centered with relatively small sample sizes. Therefore, the generalizability of our findings to other populations and regions may be limited.

Finally, some potential risk factors (such as total protein, hyperlipidemia, extubation time, and intraoperative sedation depth) could not be quantitatively analyzed due to the insufficient number of eligible studies.

## Data Availability

The original contributions presented in the study are included in the article/supplementary material, further inquiries can be directed to the corresponding author/s.
